# SD‐36 promotes growth inhibition and induces apoptosis via suppression of Mcl‐1 in glioma

**DOI:** 10.1111/jcmm.16754

**Published:** 2021-07-21

**Authors:** Shiqi Kong, Xinbo Ge, Xin Li, Zhenbo Liu, Rui Zhang, Ming Yang, Zhenhai Wang, Zhenzhong Li

**Affiliations:** ^1^ Department of Neurosurgery Xingtai People’s Hospital Hebei Province China; ^2^ Department of Neurosurgery The First People's Hospital of Shenyang Shenyang China

**Keywords:** glioma, Mcl‐1, SD‐36, STAT3, TMZ

## Abstract

Glioma is one of the most commonly observed tumours, representing approximately 75% of brain tumours in the adult population. Generally, glioma therapy includes surgical resection followed by radiotherapy and chemotherapy. The transcription factor STAT3 (signal transducer and activator of transcription 3) is a promising target for the treatment of cancer and several other diseases. At nanomolar concentrations, SD‐36 induces rapid cellular degradation of STAT3 but cannot degrade other STAT proteins. The current study demonstrates the therapeutic efficacies of the STAT3 degraders SD‐36 against glioma, as well as understanding the elucidating mechanisms and identifying molecular markers that determine cell sensitivity to STAT3 degraders. Glioma cell lines possessed similar response patterns to SD‐36 but different responses to the STAT3 inhibitor Stattic. SD‐36 potently induced apoptosis in glioma cells along with a reduction in Mcl‐1 levels, which are critical for mediating the induction of apoptosis and enhancing TMZ‐induced apoptosis. Accordingly, SD‐36 sensitizes the antitumour effect of TMZ in patient‐derived xenograft. In addition, the downregulation of Mcl‐1 expression‐mediated antitumour effect of SD‐36 was analysed in cell‐derived xenograft. These observations need to be validated clinically to confirm the efficacy of STAT3 degraders in glioma.

## 1 INTRODUCTION

In adults, the most common primary tumour of the central nervous system is glioblastoma.^1^ Even after treatments such as radiotherapy, maximal surgical resection, and chemotherapy using temozolomide (TMZ), the overall survival (OS) of patients is still only 14.6 months.^2^, ^3^ In the last three decades, while various new clinical trials conducted emerged, only a few advances have been made in treatment options.^1^, ^4^

The transcription factor STAT3 (signal transducers and activators of transcription) participates in essential processes of the cell, such as proliferation, angiogenesis, and survival, the hallmarks of initiation of oncogenicity.^5^ STAT3 is a typically expressed oncogene and regulates the expression of various downstream oncogenes, including that of itself.^6^, ^7^ In addition to phosphorylation of canonical tyrosine 705 in JAK‐STAT3 (Janus kinase‐STAT3) signalling, it mediates and facilitates cancer metastasis and growth by signalling through serine 727 phosphorylation noncanonically, and in its state with no unphosphorylation.^8^ Oncogenesis is promoted by macroenvironment inflammatory signals and the tumour microenvironment and sustained via the expression and release of factors that facilitate survival, including IL‐6 (interleukin‐6),^8^, ^9^ thereby creating an effective loop of endless feedback of autocrine and paracrine signalling to initiate and sustain reprogramming of metabolism and stalling apoptosis. Moreover, interactions of STAT3 and supercomplexes of transcription factors facilitate signalling pathways of oncogenes.^5^, ^10^

The PROTAC (PROteolysis TArgeting Chimera) technique was found during the identification of peptides or small chemical ligands that particularly bind with endogenous E3 ligases, including FBXW1A (F‐box/WD repeat‐containing protein 1A, also called b‐TRCP1), MDM2 (mouse double minute 2 homolog), the von Hippel‐Lindau tumour suppressor, and CRBN (cereblon).^11^, ^12^ PROTAC is structurally a small molecule consisting of two functional parts, a highly specific ‘warhead’ towards the POI (binding protein of interest) and a second part that acts as a ligand recognized by the E3 ligase and is connected via a linker.^13^ PROTACs provide an alternate approach for targets that may be undruggable, including transcription factors.^14^ SD‐36, one of the STAT3 degraders, possibly induces in vitro and in vivo STAT3 protein degradation and exhibits high selectivity compared with other STAT members.^15^ SD‐36 inhibits the growth of acute myeloid leukaemia and anaplastic large‐cell lymphoma cell lines,^16^ although its effect on glioma is still elusive.

In the current study, we compared the effects of SD‐36 and Stattic on the growth of glioma cells and demonstrated their therapeutic mechanisms.

## 2 MATERIALS AND METHODS

### 2.1 Cell culture

The cell lines U251 and U87 from humans were procured from the ATCC (Manassas, VA) and grown in RPMI 1640 medium along with foetal bovine serum (5%) in humid air with 5% CO_2_ at 37℃. Stable Mcl‐1 overexpression was achieved by infecting a given cell line for 24 hours with lentiviruses carrying empty vector or Mcl‐1 as described previously.^17^, ^18^ On receiving the cells, annual detection of mycoplasma was performed using the Mycoplasma Detection Kit Nozan MycoAlert (Sigma, USA) to make sure that they were free from any mycoplasma. SD‐36 and Temozolomide (TMZ) were obtained from MedChemExpress. S63845 was purchased from Chemietek (Indianapolis, IN).

### 2.2 Cell viability assay

Cell viability was analysed as previous study.^18^ Seeding of cells was done in culture 96‐well plate for 24 hours and treated with the test agents. The MTS assay was used to estimate the viable cell number as mentioned earlier. Calculation of CI (combination index) was done for drug interaction using CompuSyn software (ComboSyn, Inc; Paramus, NJ).

### 2.3 Matrigel invasion assay

Transwell chambers in 24‐well plates were got from ThermoFisher (USA) and used to perform the invasion assay. Matrigel was used to coat the Transwell chamber (BD Bioscience, USA). Cells were trypsinized 24 hours following SD‐36 treatment and were then incubated in the top chamber for 24 hours without serum. The medium supplemented with serum (10%) was placed in the bottom chamber. Non‐invaded cells were scraped off. Cells that invaded through the filter were fixed with 4% paraformaldehyde and stained with haematoxylin.

### 2.4 Apoptosis

The apoptosis detection kit with annexin V/PI from BD Biosciences (San Jose, CA) was used to detect apoptosis. Further, the ELISA kit for Cell Death Detection from Roche Diagnostics (Indianapolis, IN) was used to determine DNA fragments associated with histones as per the provided instructions. Western blotting was carried out to determine apoptosis and protein cleavage.

### 2.5 Western blotting

Western blotting was performed following previous studies.^19^, ^20^ In brief, glioma cells were lysed for 20 minutes using RIPA (radioimmunoprecipitation assay) buffer with protease inhibitors on ice and then centrifuged for 60 minutes at 20 000 *g* and 4℃. Then, the BCA assay using a Pierce BCA protein assay kit from Thermo Scientific, Inc, was carried out to determine the protein content in the separated supernatants. Fifty micrograms of protein were resolved on a 10% PAGE gel and electronically transferred onto nitrocellulose membranes for 60 minutes using a Bio‐Rad Trans‐Blot (both from Bio‐Rad Laboratories). Blocking buffer (0.05% PBS and 1% skim milk) was used on the membrane for 60 minutes at room temperature, followed by the addition of primary antibodies for one full day at 4℃. The membrane was washed using washing buffer (0.05% PBS and Tween‐20), and a secondary antibody conjugated with HRP (horseradish peroxidase) was carried out for 60 minutes at room temperature. This step was followed by three washes using wash buffer, treatment with the western blotting detection reagent ECL Prime (GE Healthcare), and observation with an LAS‐4000 system (GE Healthcare). The primary antibodies are listed as follows: cleaved caspase‐3 (#9661, Cell Signaling Technology, 1:2000), cleaved caspase‐8 (#9496, Cell Signaling Technology, 1:1000), cleaved PARP (#9148, Cell Signaling Technology, 1:1000), STAT3 (ab68153, Abcam, 1:2000), Bcl‐xL (ab32370, abcam, 1:1000), PUMA (#4976, Cell Signaling Technology, 1:1000), DR5 (ab8416, abcam, 1:2000), Mcl‐1 (sc‐12756, Santa Cruz biotechnology, 1:2000), Bcl‐2 (#3498, Cell Signaling Technology, 1:1000), β‐actin (A5441, Sigma, 1:5000), E‐cadherin (#3195, Cell Signaling Technology, 1:1000), N‐cadherin (#13116, Cell Signaling Technology, 1:1000), vimentin (#5741, Cell Signaling Technology, 1:1000), snail (#4719, cell signaling technology, 1:1000).

### 2.6 Real‐time PCR

Real‐time PCR was performed following previous studies.^21^, ^22^ Total cellular RNA was isolated using TRIzol reagent from Sigma Chemical according to the provided instructions and reverse‐transcribed using the reverse transcriptase SuperScript III from Promega (Madison, WI). Real‐time PCR was performed using the PCR Master Mix Reagent SYBR Green and the 7500 Fast real‐time PCR system from Applied Biosystems (Carlsbad, CA). The conditions for amplification were as follows: 2 minutes at 50℃ and 10 minutes at 95℃, followed by 40 cycles of 15 seconds at 95℃ and 60 seconds at 60℃. The negative controls were reactions without the template or reverse transcriptase. The internal control was GAPDH (glyceraldehyde 3‐phosphate dehydrogenase) for normalization. The primers are listed as follows: Mcl‐1: Forward: 5′‐GGACACAAAGCCAATGGGCAGGT‐3′, Reverse: 5′‐GCAAAAGCCAGCAGCACATTCCTGA‐3′; GAPDH: Forward: 5′‐CTGCCGTCTAGAAAAACC‐3′, Reverse: 5′‐CCAAATTCGTTGTCATACC‐3′.

### 2.7 Small interfering RNA (siRNA)‐mediated gene knockdown

STAT3 (CAUCUGCCUAGAUCGGCUAdTdT) and Mcl‐1 (CGAAGGAAGUAUCGAAUUUdTdT) siRNAs were obtained from Santa Cruz Biotechnology, Inc Transfection of siRNAs was performed with Lipofectamine 3000 from Invitrogen according to the provided instructions.

### 2.8 PDX mouse model

Approval for experiments on animals was obtained from the Animal Care and Use Committee of Xingtai People's Hospital. The PDX mouse model was established with treatment‐free primary tumours obtained from patients with glioma. Briefly, tissues were cut into 30 mg pieces and implanted subcutaneously into both flanks of NOD. Cg‐*Prkdc^scid^ Il2rg^tm1Wjl^*/SzJ (NSG) mice. Mice were treated with SD‐36 (2 mg/kg/d; twice/week, ip), TMZ (10 mg/kg/d; twice/week, ip), or a combination. Callipers were used to measure tumour volumes and determined using the formula V = ½ × L × W^2^, where V is tumour volume, W is tumour width, and L is tumour length. As the treatment ended, the weights of the mice were determined, and the mice were euthanized by CO_2_ asphyxiation. The tumours were then removed, weighed, and frozen in liquid nitrogen for further analyses.

### 2.9 Xenograft

Approval for experiments on animals was obtained from the Xingtai People's Hospital Institutional Animal Care and Use Committee (IACUC). For treatments, SD‐36 (5 mg/kg/d; twice/week, ip) was used. Callipers were used to measure tumour volumes and determined using the formula V = 1/2 × L×W^2^. As the treatment ended, the weights of the mice were determined and euthanized by CO_2_ asphyxiation. The tumours were then removed, weighed, and frozen in liquid nitrogen for further analyses.

### 2.10 Immunohistochemical (IHC) staining

Antigen extraction was performed using 0.01 mol/L citrate buffer after pre‐treatment of the sections with xylene and rehydration through a graded alcohol series. Background activity was removed with hydrogen peroxide. Goat Serum (Sigma‐Aldrich, USA) was then used to treat the tissue sections for 30 minutes to block non‐specific binding. Afterward, the indicated primary antibodies were added and the slides were incubated overnight at 4℃. After DAB staining, counterstained the slices with haematoxylin, and mounted in neutral gum to observe the paraffin sections. The tissues were then analysed by a bright‐field microscope.

### 2.11 Statistical analysis

Two‐sided unpaired Student's t tests analysed the statistical significance of variations between two groups in terms of tumour sizes or weights in the case of equal variances. The same software was used to examine the data to confirm the assumptions for using the t tests. One‐way ANOVA assessed the differences between multiple treatments. At *P* < .05, outcomes were statistically significant.

## 3 RESULTS

### 3.1 SD‐36 suppresses the cell growth, invasion and induces apoptosis of human glioma cell lines

**FIGURE 1:**
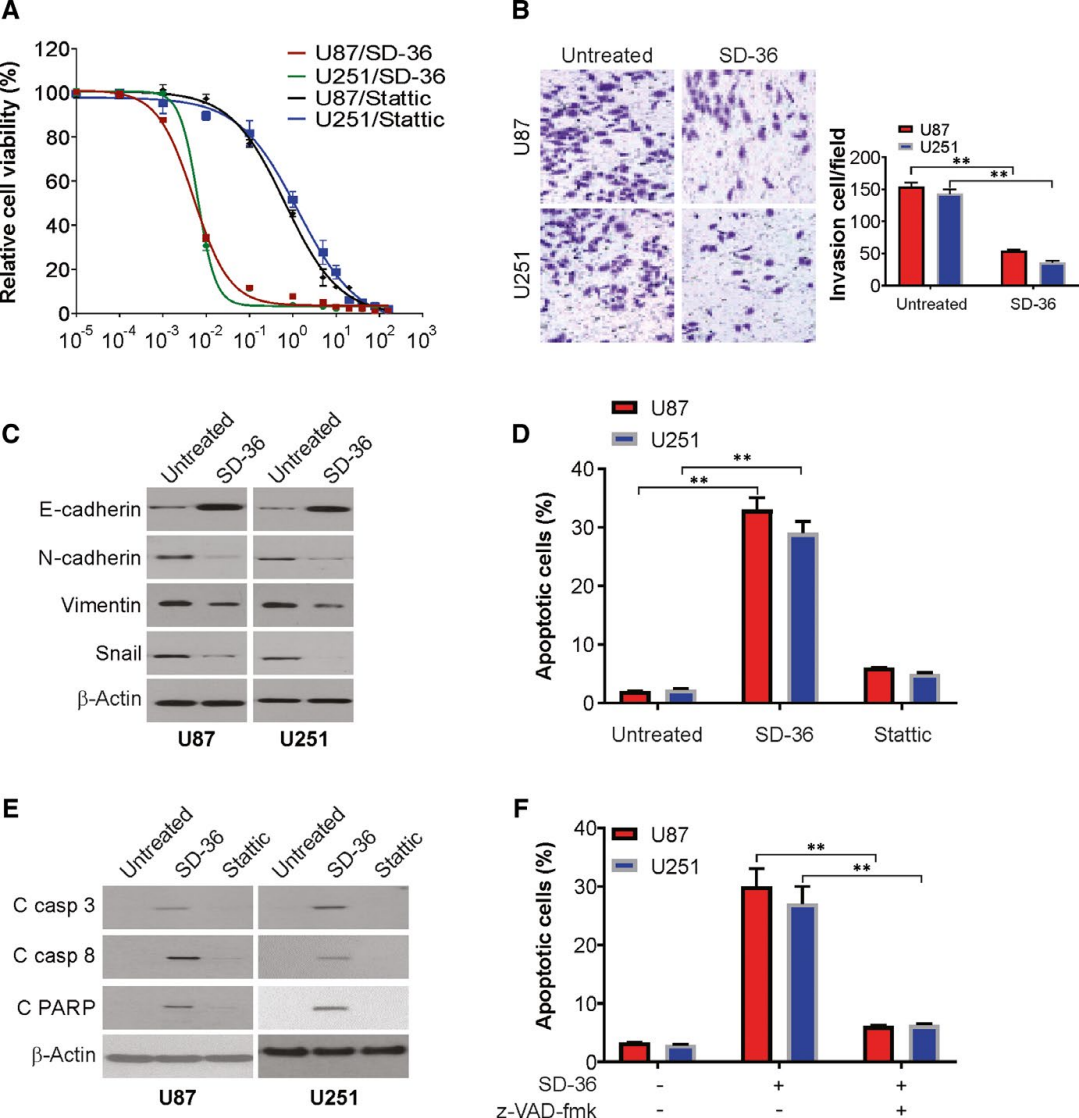
SD‐36 induces apoptosis in glioma. A, U87 and U251 cells were treated with increasing concentration of SD‐36 or Stattic for 72 h. Cell viability was analysed by MTS assay. B, Invasion of U87 and U251 cells treated with SD‐36 (100 nmol/L) was analysed by Transwell assay. C, U87 and U251 cells were treated with 100 nmol/L SD‐36 or 1 μmol/L Stattic for 24 h. Indicated protein levels were analysed by western blotting. D, U87 and U251 cells were treated with 100 nmol/L SD‐36 or 1 μmol/L Stattic for 24 h. Apoptosis was analysed by flow cytometry. E, U87 and U251 cells were treated with 100 nmol/L SD‐36 or 1 μmol/L Stattic for 24 h. Indicated protein levels were analysed by western blotting. F, U87 and U251 cells were treated with 100 nmol/L SD‐36 for 24 h with or without z‐VAD‐fmk pre‐treatment. Apoptosis was analysed by flow cytometry. Data are presented as the mean ± SD from three independent experiments, **P < .01

We first tested the responses of U87 and U251 glioma cell lines to the STAT3 degrader SD‐36 and Stattic using a 3‐day cell viability assay and found that the survival rate of these cell lines decreased more strongly with SD‐36 in general than Stattic treatment (Figure 1A). Next, we determined the effects of SD‐36 on invasion in glioma cells. As shown in Figure 1B, SD‐36 treatment decreases cell invasion in U87 and U251 cells, which was detected by Transwell assay. Furthermore, the effect of SD‐36 on epithelial‐mesenchymal transition (EMT) was detected by western blotting. Our findings revealed that SD‐36 treatment resulted in increase in E‐cadherin and decrease in N‐cadherin, vimentin and snail in U87 and U251 cells (Figure 1C).

To determine whether SD‐36 suppresses the growth of glioma cells via apoptotic induction, we next compared the effects of SD‐36 and Stattic on apoptotic induction in 2 glioma cell lines. As presented in Figure 1D, SD‐36 at 100 nmol/L effectively enhanced apoptotic populations in the two cell lines. Stattic at 1 μmol/L did not apparently induce apoptosis in these cell lines. Similarly, PARP and caspase cleavage results showed that SD‐36, but not Stattic, under the same tested conditions effectively induced caspase‐8, caspase‐3, and PARP cleavage, primarily in the cells (Figure 1E). Moreover, SD‐36‐induced apoptosis was blocked by z‐VAD‐fmk pre‐treatment (Figure 1F). Together, these results indicate that the tested STAT3 degrader SD‐36 possesses more potent activities than Stattic in inducing apoptosis of human glioma cells.

### 3.2 SD‐36 effectively decreases STAT3 levels and exerts different effects from Stattic on the modulation of Mcl‐1 levels in human glioma cells

**FIGURE 2:**
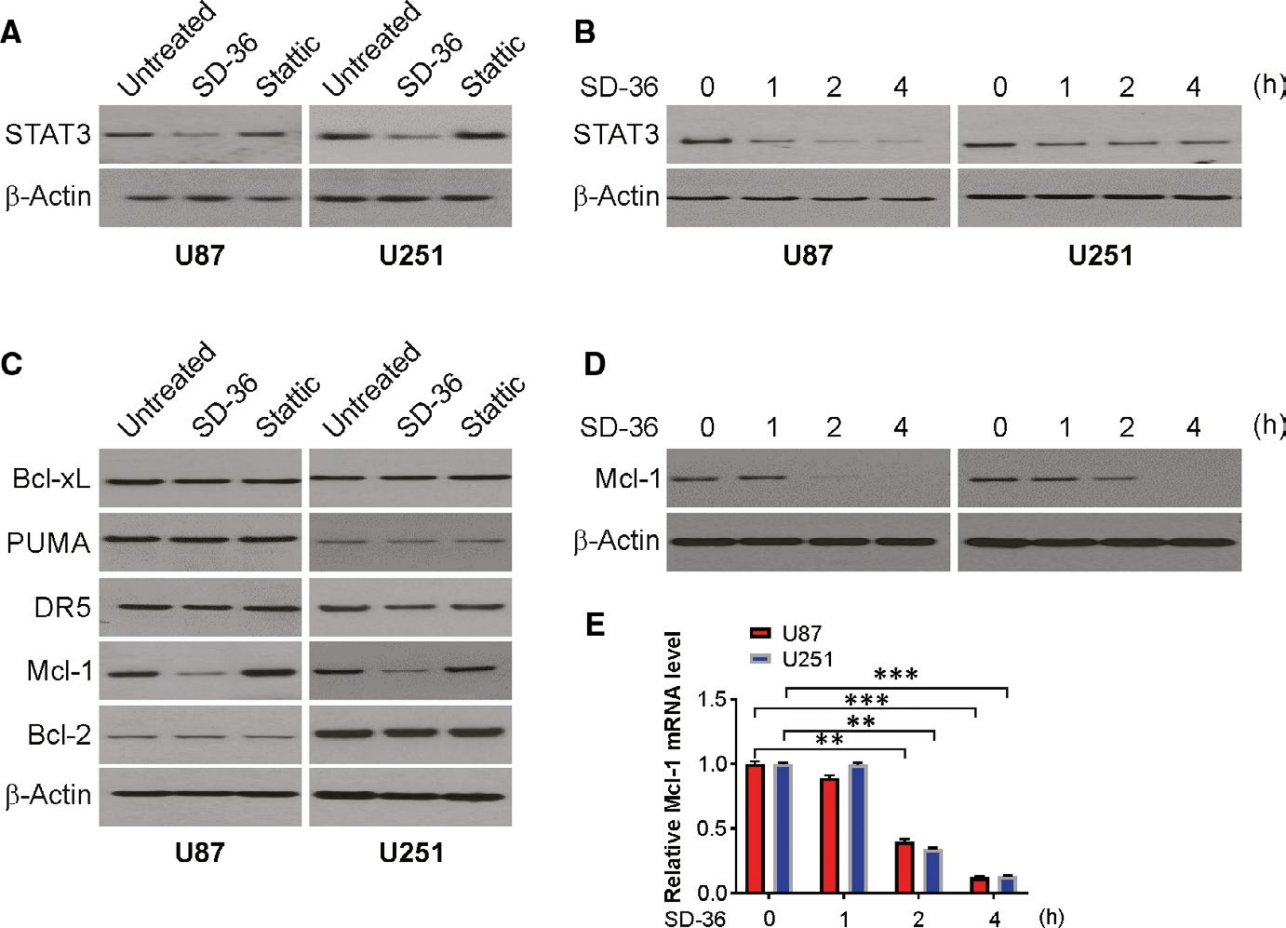
SD‐36 downregulates Mcl‐1 level. A, U87 and U251 cells were treated with 100 nmol/L SD‐36 or 1 μmol/L Stattic for 24 h. STAT3 was analysed by western blotting. B, U87 and U251 cells were treated with 100 nmol/L SD‐36 at indicated time points. STAT3 was analysed by western blotting. C, U87 and U251 cells were treated with 100 nmol/L SD‐36 or 1 μmol/L Stattic for 24 h. Indicated protein levels were analysed by western blotting. D, U87 and U251 cells were treated with 100 nmol/L SD‐36 at indicated time points. Mcl‐1 was analysed by western blotting. E, U87 and U251 cells were treated with 100 nmol/L SD‐36 at indicated time points. mRNA level of Mcl‐1 was analysed by real‐time PCR. Data are presented as the mean ± SD from three independent experiments, **P < .01

To understand the mode of action by which STAT3 degrader SD‐36 induce apoptosis in glioma cells, we assessed the effects of SD‐36 compared to Stattic on the modulation of multiple proteins related to STAT3 signalling and apoptosis primarily in U87 and U251 cell lines. We found that SD‐36, but not Stattic, decreased STAT3 levels in the examined glioma cell lines (Figure 2A); these events occurred very rapidly even at 1 hour post‐treatment (Figure 2B). Hence, SD‐36 indeed effectively induced degradation of STAT3 protein, its putative target, in human glioma cells.

In addition, we examined the role of these agents in the modulation of several proteins participating in apoptotic regulation in U251 and U87 cells. SD‐36 minimally affected the Bcl‐X_L_, Bcl‐2, PUMA, and DR5 levels in the tested cell lines. Nevertheless, SD‐36 effectively reduced the level of Mcl‐1 in U87 and U251 cell lines, whereas Stattic did not decrease the Mcl‐1 level at the tested concentrations in U87 and U251 cells (Figure 2C). Time‐course analyses showed an apparent decline in the expression of Mcl‐1 2 hours or more after treatment after a reduction in STAT3 protein expression (Figure 2D), suggesting that the change in Mcl‐1 is likely secondary to STAT3 degradation.

We next determined the mechanisms by which SD‐36 induces a reduction in Mcl‐1 levels in glioma cell lines. Given that STAT3 proteins function primarily as regulators of gene transcription, we first examined the mRNA levels of Mcl‐1 in U87 cells exposed to SD‐36 for various times and found that SD‐36 treatment significantly reduced the mRNA levels of Mcl‐1 over treatment times spanning 1‐4 hours (Figure 2E). A reduction in Mcl‐1 mRNA was also observed in the U251 cells exposed to SD‐36.

### 3.3 Mcl‐1 suppression is required for SD‐36‐induced apoptosis in glioma cells

**FIGURE 3:**
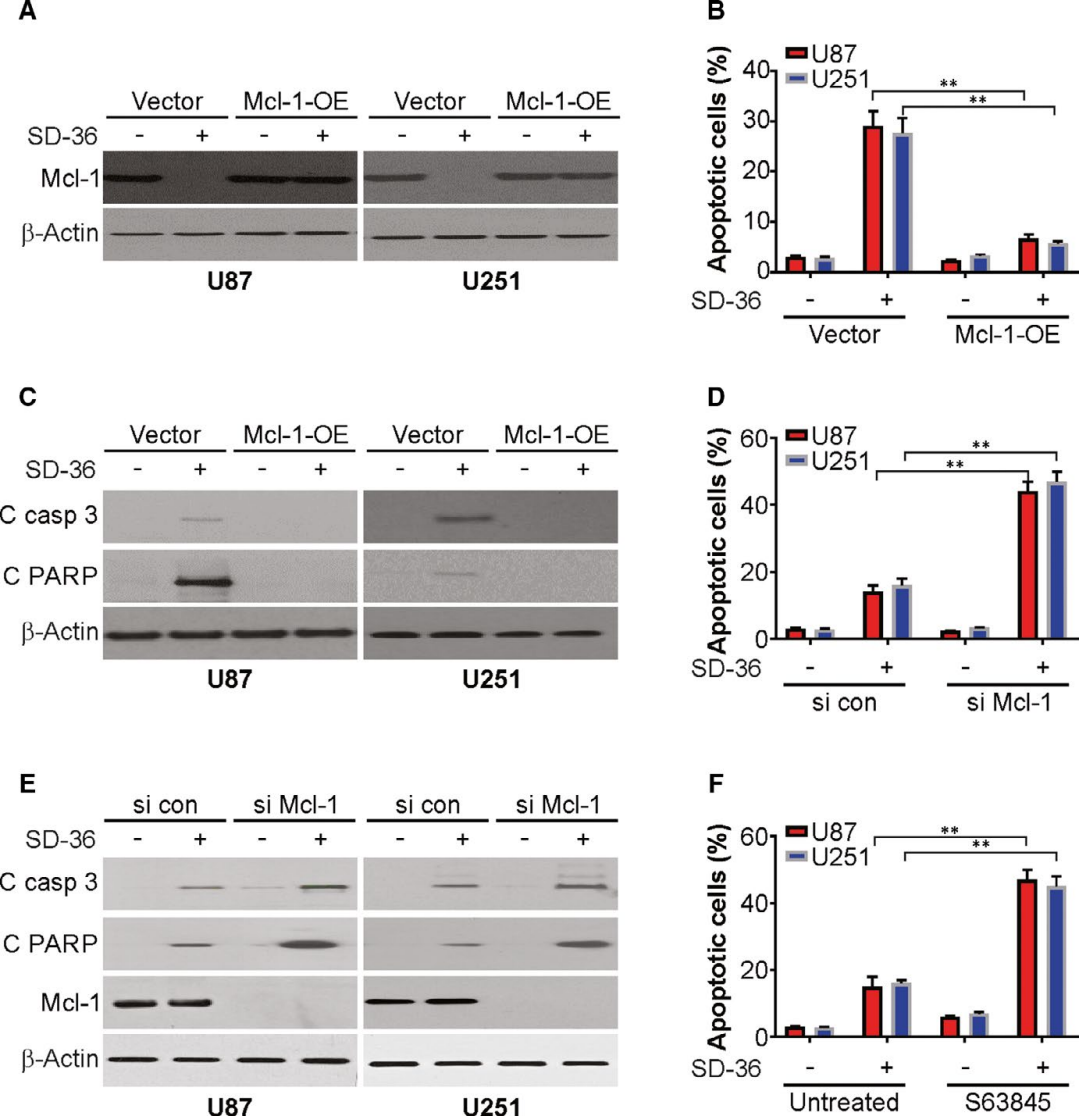
Mcl‐1 downregulation is required for SD‐36‐induced apoptosis. A, U87 and U251 cells transfected with Mcl‐1 were treated with 100 nmol/L SD‐36 for 24 h. Mcl‐1 was analysed by western blotting. B, U87 and U251 cells transfected with Mcl‐1 were treated with 100 nmol/L SD‐36 for 24 h. Apoptosis was analysed by flow cytometry. C, U87 and U251 cells transfected with Mcl‐1 were treated with 100 nmol/L SD‐36 for 24 h. Indicated protein levels were analysed by western blotting. D, U87 and U251 cells transfected with si RNA against Mcl‐1 were treated with 10 nmol/L SD‐36 for 24 h. Apoptosis was analysed by flow cytometry. E, U87 and U251 cells transfected with si RNA against Mcl‐1 were treated with 10 nmol/L SD‐36 for 24 h. Indicated protein levels were analysed by western blotting. F, U87 and U251 cells were treated with 10 nmol/L SD‐36, 1 μmol/L S63845, or their combination for 24 h. Apoptosis was analysed by flow cytometry. Data are presented as the mean ± SD from three independent experiments, **P < .01

To demonstrate the effect of suppression of Mcl‐1 on apoptotic induction by SD‐36, we induced ectopic expression of Mcl‐1 in U87 and U251 cells and then analysed their responses to SD‐36. To this end, we transiently expressed Mcl‐1 by infecting the tested cell lines with lentiviruses carrying empty vector and Mcl‐1 and then exposed these cells to DMSO or SD‐36 for 24 hours. Mcl‐1 overexpression attenuated SD‐36‐induced downregulation of Mcl‐1 expression (Figure 3A). We detected remarkably less apoptosis in both U87 and U251 cells expressing ectopic Mcl‐1 than in the corresponding vector control cells (Figure 3B,C), indicating that induced Mcl‐1 expression indeed protected cells against apoptosis induced by SD‐36. Complementarily, we knocked down Mcl‐1 in U87 cells and then exposed them to low‐dose SD‐36 and found that knockdown of Mcl‐1 significantly sensitized U87 cells to apoptosis (Figure 3C,D). Similarly, the combination of SD‐36 with S63845, a Mcl‐1 inhibitor, enhanced apoptotic induction in U251 and U87 cell lines in comparison with low‐dose SD‐36 or S63485 alone (Figure 3E,F). Therefore, the above data suggest that downregulation of Mcl‐1 expression is required for SD‐36‐promoted apoptosis.

### 3.4 SD‐36 synergizes with TMZ to enhance apoptosis

**FIGURE 4:**
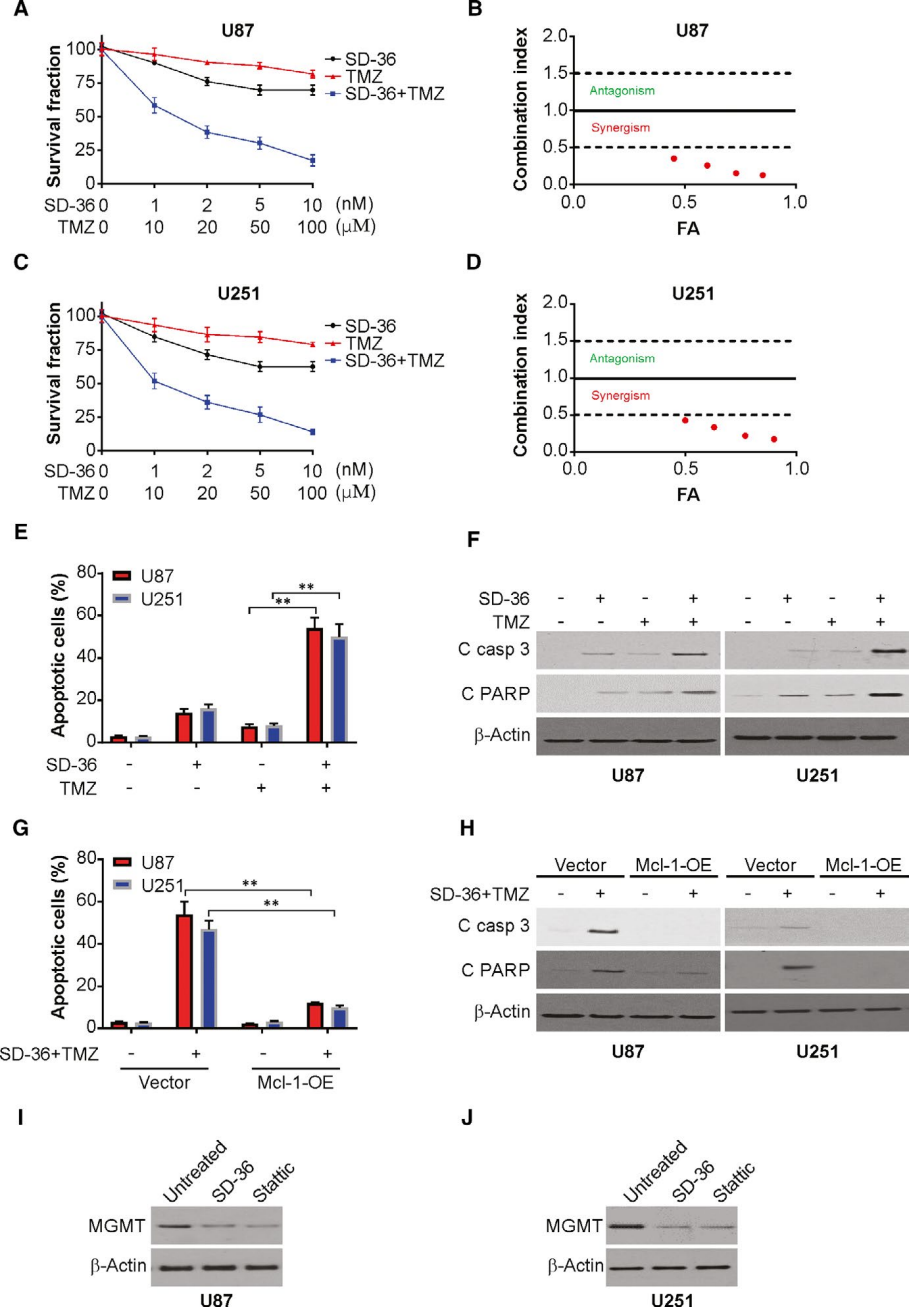
SD‐36 sensitizes TMZ‐induced apoptosis in glioma. A, U87 cells were treated with SD‐36 and TMZ at indicated concentration for 24 h. Cell viability was analysed by MTS. B, Combination index is shown for U87 cells. FA, fraction affected. C, U251 cells were treated with SD‐36 and TMZ at indicated concentration for 24 h. Cell viability was analysed by MTS. D, Combination index is shown for U251 cells. FA, fraction affected. E, U87 and U251 cells were treated with 5 nmol/L SD‐36, 50 μmol/L TMZ, or their combination for 24 h. Apoptosis was analysed by flow cytometry. F, U87 and U251 cells were treated with 5 nmol/L SD‐36, 50 μmol/L TMZ, or their combination for 24 h. Indicated protein levels were analysed by western blotting. G, U87 and U251 cells transfected with Mcl‐1 were treated with the combination of 5 nmol/L SD‐36 and 50 μmol/L TMZ for 24 h. Apoptosis was analysed by flow cytometry. H, U87 and U251 cells transfected with Mcl‐1 were treated with the combination of 5 nmol/L SD‐36 and 50 μmol/L TMZ for 24 h. Indicated protein levels were analysed by western blotting. I, U87 cells were treated with 100 nmol/L SD‐36 or 1 μmol/L Stattic for 24 h. MGMT protein level was analysed by western blotting. J, U251 cells were treated with 100 nmol/L SD‐36 or 1 μmol/L Stattic for 24 h. MGMT protein level was analysed by western blotting. Data are presented as the mean ± SD from three independent experiments, **P < .01

We next determined whether SD‐36 enhances apoptosis when combined with TMZ. In both U251 and U87 cells, TMZ and SD‐36 combined led to a further decrease in cell survival with IC50 values of <1, indicating synergistic effects (Figure 4A‐D). In agreement, the combinations of TMZ with SD‐36 showed significantly stronger effects than either agent alone in enhancing cell populations positive for annexin V (Figure 4E) and in augmenting cleavage of PARP and caspase‐3 and caspase‐8 (Figure 4F). However, the enhanced effects of the SD‐36 and TMZ combination on apoptosis, including PARP cleavage, were abolished in cells highly expressing ectopic Mcl‐1 (Figure 4G,H), indicating the decline in Mcl‐1 expression is critical in facilitating this event. Previous study has shown STAT3 inhibition overcomes TMZ resistance by reducing MGMT expression in glioma.^23^ Therefore, we detected the effect of SD‐36 on MGMT expression in glioma. As shown in Figure 4I,J. SD‐36, as well as Stattic, decrease of MGMT protein level in U87 and U251 cells, which indicate that MGMT downregulation may contribute to the synergistic effect of SD‐36 TMZ in glioma cells.

### 3.5 SD‐36 sensitizes TMZ‐induced apoptosis in PDX mouse model

**FIGURE 5:**
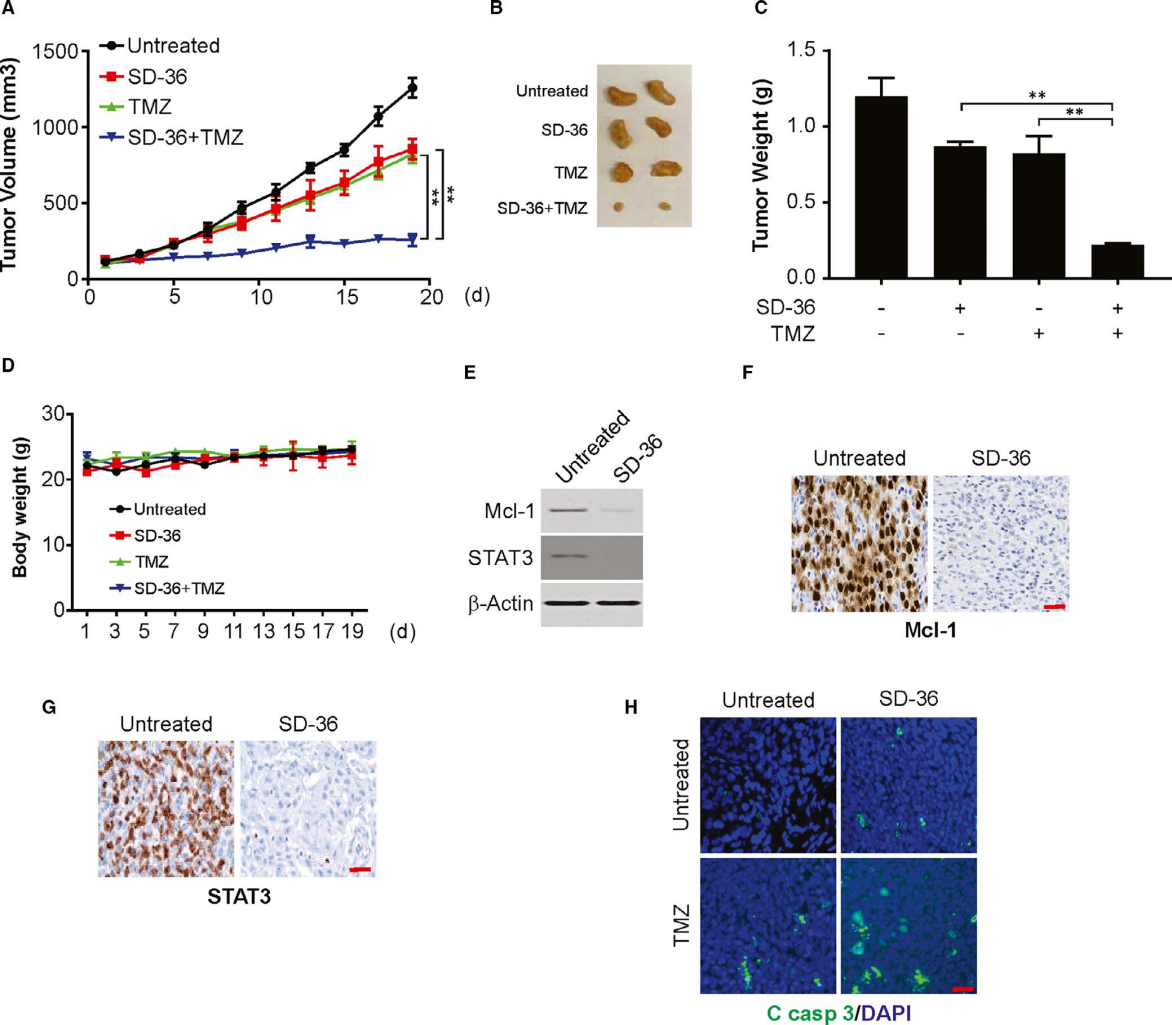
SD‐36 enhances the antitumour effect of TMZ in PDX model. A, Tumour volume quantification of established PDX tumours in NSG mice treated with vehicle, SD‐36, TMZ or the combination of SD‐36 and TMZ. B, Representative tumours at the end of the experiment in (A). C, Tumour weight of established PDX tumours in NSG mice treated with vehicle SD‐36, TMZ or the combination of SD‐36 and TMZ. D, Bodyweight of tumour‐bearing mice treated with vehicle, SD‐36, TMZ, or the combination of SD‐36 and TMZ. E, Western blotting of Mcl‐1 in vehicle‐treated and SD‐36‐treated PDX tumours. F, Mcl‐1 expression level was analysed in the indicated tumours by IHC. Scale bars: 25 μm. G, STAT3 expression level was analysed in the indicated tumours by IHC. Scale bars: 25 μm. H, Cleaved caspase‐3 immunostaining of PDX tumours. Scale bars: 25 μm. Data are presented as the mean ± SD from three independent experiments, **P < .01

The effect of SD‐36 and TMZ in the PDX model was evaluated. Each single agent was compared against the SD‐36 and TMZ combination in the model (Figure 5A‐C). The combination was found to exhibit a substantially stronger effect than either agent alone in the PDX models. However, the mouse bodyweight was not altered by the combined treatment (Figure 5D). In addition, SD‐36 treatment decreased Mcl‐1 and STAT3 expression in PDX tumours (Figure 5E‐G). Cotreatment induced more apoptosis in tumours than single treatment (Figure 5H). The above data indicate that SD‐36 sensitizes TMZ in the PDX model.

### 3.6 Effective inhibition of human glioma xenograft growth by SD‐36

**FIGURE 6:**
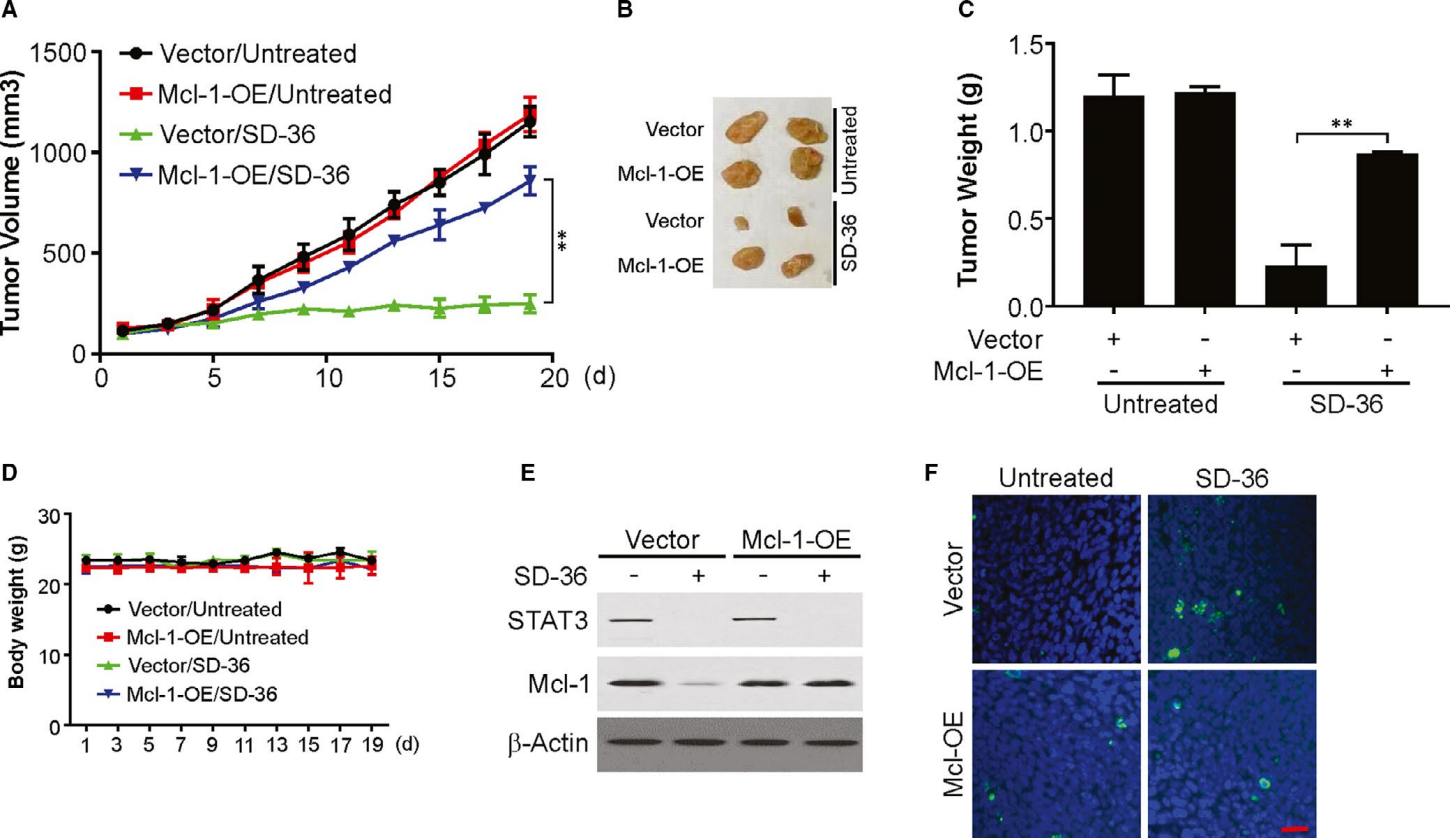
Mcl‐1 downregulation is required for the antitumour effect of SD‐36 in vivo. A, Nude mice were injected s.c. with 5 × 10^6^ Vector or Mcl‐1‐OE U87 cells. After 7 d, mice were treated with 10 mg/kg SD‐36 or the vehicle control for 10 consecutive days. Tumour volume at indicated time points after treatment was calculated and plotted with *P* values, n = 6 in each group. B, Representative tumours at the end of the experiment in (A). C, Tumour weight of indicated groups. D, Bodyweight of tumour‐bearing mice treated with vehicle or SD‐36. E, Western blotting of Mcl‐1 and STAT3 in vehicle‐treated and SD‐36‐treated tumours. F, Cleaved caspase‐3 immunostaining of indicated tumours treated with SD‐36 for 4 consecutive days. Scale bars: 25 μm. Data are presented as the mean ± SD from three independent experiments, **P < .01

Next, we investigated the effect of SD‐36 on the growth of parental and Mcl‐1‐overexpressing (Mcl‐1‐OE) U251 xenografts. Treatment with SD‐36 at 5 mg/kg significantly impeded the growth of WT U251 xenografts, which was attenuated in Mcl‐1‐OE tumours (Figure 6A‐C). Under the same tested conditions, SD‐36 did not reduce mouse bodyweights, suggesting no toxicities at this concentration (Figure 6D). By analysing several protein markers altered by SD‐36, we detected significantly reduced levels of STAT3 and Mcl‐1 in the SD‐36‐treated U251 tumours (Figure 6E). However, Mcl‐1‐OE attenuated SD‐36‐induced apoptosis (Figure 6F). Thus, SD‐36 effectively impedes the growth of glioma xenografts by reducing STAT3 and Mcl‐1 levels and enhancing apoptosis in vivo.

## 4 DISCUSSION

A common cancer type of the central nervous system, glioma, is a primary reason for deaths due to cancer because of high‐grade invasion and growth of glioma cells.^24^ Treatment for glioma patients includes radiotherapy, surgery, chemotherapy, and a combination of these.^25^ Furthermore, patients often have a poor prognosis with a 5‐year survival rate of nearly 10%.^26^ Clinically, a common drug for chemotherapeutic glioma treatment is TMZ.^27^ Nevertheless, TMZ resistance is often observed during treatment.^3^, ^28^ Hence, it is imperative to discover new agents for better outcomes by examining the mode of occurrence and progression of glioma.

The current study has clearly shown that the novel STAT3 degrader SD‐36^15^ exhibits much more potent activity than Stattic in decreasing the survival of a panel of human cell lines for glioma, in apoptotic induction of these cells and in inhibiting the growth of glioma xenografts and PDXs in vivo. Our results suggest that STAT3 degraders and conventional STAT3 inhibitors may function through different, in addition to overlapping, mechanisms despite all targeting STAT3 proteins. Although STAT3 degraders and inhibitors all target STAT3 to exert their anticancer activities, it was unclear whether the presence and abundance of STAT3 protein in cancer cells correlate with or predict cell sensitivities or responses to these STAT3‐targeting agents.

STAT3 has several biological functions and is a member of the STAT family proteins.^7^ Upon stimulation with several growth factors and cytokines, tyrosine kinases such as JAK are activated, causing phosphorylation of STAT3 Tyr705.^29^ Then, a dimer of phospho‐STAT3 forms via the SH2 domain's interaction with the phospho‐Tyr motif, causing nuclear translocation and binding to specific DNA sequences for target gene transcriptional activation.^30^ Tumour proliferation and progression are promoted by activated STAT3 via gene expression regulation participating not only in cancer cell invasion and survival but also in immune escape and angiogenesis in the microenvironment of the tumour.^31^ Constitutive activation of STAT3 occurs in solid and haematologic tumours, but activation is transient in normal cells.^5^ Therefore, STAT3 is a potentially attractive target for cancer treatment.

In our study, we describe the novel finding that SD‐36 decreased Bim levels beyond their effects on reducing Mcl‐1 levels in sensitive human glioma cells, whereas Stattic clearly increased Bim levels in these cell lines. Here, we demonstrated that SD‐36 induces apoptosis involving suppression of Mcl‐1 in human glioma cancer cells since induced ectopic Mcl‐1 expression remarkably attenuated apoptosis induced by SD‐36 in U87 and U251 cells, whereas Mcl‐1 knockdown or inhibition enhanced apoptosis induced by SD‐36 in glioma cells. Bim reduction seems to have no negative impact on SD‐36‐induced apoptosis because deficiency of Bim in these cell lines, generated with CRISPR/Cas 9 gene knockout technology, did not compromise the capacity of SD‐36 to induce apoptosis although it did safeguard the cells from apoptotic induction by Stattic. In summary, SD‐36 induces Bim‐independent apoptosis in glioma cells.

The molecules related to TMZ resistance in GBM include MGMT (O6‐methylguanine‐DNA methyltransferase), MPG (N‐methylpurine DNA glycosylase), and HIF‐1α (hypoxia‐inducible factor 1α).^3^ This resistance against TMZ is mediated by enhancing MGMT levels.^32^ In particular, the methylation of the MGMT promoter is a predictive biomarker associated with alkylating chemotherapy, especially TMZ.^33^ Various other mechanisms of action recently associated with TMZ resistance have been observed.^34^ For instance, downregulating HIF‐1α expression increased the sensitivity of GBM cells to TMZ.^35^ In GBM, hedgehog (Hh) signalling is deregulated and correlates with TMZ resistance.^36^ Further, connexins, especially Cx43 or the gap junction protein connexin 43, participate in the malignant glioma microenvironment and confer resistance of GBM cells to chemotherapeutic agents, such as TMZ.^37^ Recently, PI3K pathway activation was also shown to be related to TMZ resistance.^38^ Further, Mcl‐1 suppression contributed to an increase in apoptosis induced by TMZ by SD‐36 in glioma cells because induced Mcl‐1 overexpression attenuated the increased rate of apoptosis induced by the combination.

In this study, we observed that SD‐36 possessed more potent activity than Stattic in inhibiting the growth of human glioma. Downregulation of Mcl‐1 expression is necessary for SD‐36‐induced apoptosis. Moreover, SD‐36 enhances the antitumour activity of TMZ in glioma cells and PDX model. Therefore, further improvement in the compound or optimization of its dosages or administration routes to achieve better anticancer activity with acceptable safety profiles and future testing of its anticancer activity in the clinic are warranted.

## CONFLICT OF INTEREST

The authors declare no conflict of interest.

## AUTHOR CONTRIBUTIONS

**Shiqi Kong:** Data curation (equal); Investigation (equal); Methodology (equal). **Xinbo Ge:** Data curation (equal). **Xin Li:** Data curation (equal). **Zhenbo Liu:** Conceptualization (equal); Formal analysis (equal). **Rui Zhang:** Data curation (equal). **Ming Yang:** Formal analysis (equal). **Zhenhai Wang:** Formal analysis (equal). **Zhenzhong Li:** Project administration (equal).
